# Supplementary material for: Prevalence of undernutrition and its associated factors among orphans aged 6–59 months in Nekemte town, Ethiopia
Source: PLoS One. 2025 Nov 13;20(11):e0336300. doi: 10.1371/journal.pone.0336300 (PMC12614551; doi:10.1371/journal.pone.0336300)
Supplement: Supporting Information 1 — (DOCX) [file pone.0336300.s001.docx]

**ANNEX III: DATA COLLECTION TOOLS**

**WOLLEGA UNIVERSITY**

**INSTITUTE OF HEALTH SCIENCE**

**DEPARTMENT OF PEDIATRICS & NEONATAL NURSING**

**English Version Questionnaires**

Demographic and food insecurity variables, dietary intake, and anthropometric measurements, social and legal services

Please circle or write the respondent's commitment to participate in this Study

 Agree

Disagree (End the interview)

Signature of Participant: ____________________ Date _____________________

CODE OF THE QUETIONNAIRE_______________

Name of the interviewer ________________________

Signature____________________________________

Date of interview (DD/MM/yyy) _________________

Name................................ Signature.....................  Date....................

Part I: Demography and Socioeconomic Characteristics

| Code | Questions | Coding category | Skip to |
| --- | --- | --- | --- |
| 01 | Age of the child in Month | _______________month |  |
| 02 | Age of the child at the time you received in month | _______________ |  |
| 03 | Sex of the child | 1. Male  2. Female |  |
| 04 | Where does the child stay on day time? | 1. Guardian house 2. School 2. Others(specify) |  |
| 05 | From whom did you take the child? | 1. Government organization 2. NGO 3. Individual person 3. Others( specify) |  |
| 06 | Religion of caretaker | 1. Orthodox  2. Muslim  3. Protestant  4. Other, Specify___________ |  |
| 07 | Whom did the child loss? | 1. Father 2. Mother 3. Both Father and mother |  |
| 08 | The respondent’s relation with the child | 1. Parents 2. Brothers 3. Sisters 4. Grandparents 5. Other relatives  6. neighborhood |  |
| 09 | Age of the care takers/  Guardia | _____________years |  |
| 10 | Occupation of the care  taker/Guardian | 1. House wife 2. Private employee  3. Government employ 4.Merchant  5. Other, Specify_____ |  |
| 11 | Marital status of the care  taker/Guardian | 1. 1.nuclear family 2.others _____________________ |  |
| 12 | Your family daily/ monthly income sufficient for family life? | 1.Of course sufficient 2.Medium, 3 .No |  |
| 13 | Number of under Five children in HH with the child | _________________ |  |
| 14 | How many members are present in the HH now | _______________________ |  |

Part II: Sanitation and Hygiene related question

| Code | Questions | Coding category | Skip to |
| --- | --- | --- | --- |
| 15 | What is source of your drinking water? | 1. Pipe. 2. protected spring /well 2. Other, Specify __________ |  |
| 16 | What Method used for water storage? | 1. Pot 2. Jeri Can 3. Bucket 4. Other, Specify ________ |  |
| 17 | Do you wash your hands with soap and water Whenever you feed your child? | 1. Yes 2. No |  |
| 18 | Do you often wash your hand just after toilet? | 1. Yes 2. No |  |
| 19 | What did has used to wash his/her hands after visiting toilet? | 1. Only water 2. water with soap  3. others-----------------(specify) |  |

Part III Social and Legal Services

| Code | Questioners | Coding category | Skip to |
| --- | --- | --- | --- |
| 20 | Does the child have social support? | 1. Yes 2. No | If no skip to Q. 23 |
| 21 | If yes what type of organization | 1. Non-governmental organization  2.Governmental organization  3 Religious institutions |  |
| 22 | Is there any support given to the orphan? | 1. Yes 2. No | If no skip to Q.25 |
| 23 | What type of Support is Provided by other organizations (persons)? (more than one answer is possible) | 1. Nutritional support 2. HealthCare  3. Income generating activities  4. Educational support 5. Psychosocial Support 6. Legal Protection 7. Shelter and Care 8. No support provided  9. Other (specify)……………. |  |
| 24 | Does the child have legal follow- up | 1. Yes 2. No | If no skip to Q. 26 |
| 25 | If yes how frequent they follow the child | ----------------------------- |  |

Part III: Dietary intake

| Code | Questions | Coding category | Skip to |
| --- | --- | --- | --- |
| 31 | Did the child receive treatment for acute or chronic illnesses? | 1 yes 2. no |  |
| 32 | Was the child tested for malnutrition? | 1 yes 0, no |  |
| 33 | Did the child Breast feed? | 1.Yes 0.No 2.Don‟t know | If no, skip  to 44 |
| 34 | Did the child exclusively breastfeed? | 1. Yes 0’. No 2. Don’t know |  |
| 35 | For how long the child Breastfeed? | _______month |  |
| 36 | At what age did you start to give food in Addition to your breast milk? | 1.Immediately after birth 2.Within 1 to 6 months  3.Within 6 to 12 months  4.Twelve month later |  |
| 37 | What is the first food caretakers used to feed the child? | 1. Milk 2. Adult food 3. Pour age  4.Other specify |  |
| 38 | What do caretakers used to feed the child? | 1. Hand 2. Cup and spoon  3. Bottle 4. Don’t know |  |
| 39 | Did the child eat any flat bread, biscuits, or any other foods made from cereal (maize, sorghum, millet, wheat, barely or teffe) yesterday? | 1. Yes 0. No |  |
| 40 | Did the child eat any pumpkin, carrots/orange flesh sweet potatoes, potato, onion, white yam, and other foods made from roots yesterday? | 1. Yes 0. No |  |
| 41 | Did the child eat any dark green leafy vegetables (kale, Swiss chard, cabbage) and other vegetables (tomato, onion) yesterday? | 1. Yes 0. No |  |
| 42 | Did the child eat any fruits like ripe mango, papaya, banana, avocado and lemon and orange...) and other fruits yesterday? | 1. Yes 0. No |  |
| 43 | Did the child eat any flesh meat (beef, lamb, goat, chicken) and any organ meat (liver, kidney, heart) yesterday? | 1. Yes 0. No |  |
| 44 | Did the child drink milk and milk products yesterday? ( milk, cheese, yogurt or other milk products) | 1. Yes  0. No |  |
| 45 | Did the child eat any food with oil, fat or butter yesterday | 1. Yes  0. No |  |

Part IV: Health related variables

| Code | Questions | Coding category | Skip to |
| --- | --- | --- | --- |
| 46 | Do you have vaccination card | 1. Yes  0. No | If no skip to Q.56 |
| 47 | What vaccine the child taken? | 1. BCG 2. Polio/ 3.Measles  4 others________ |  |
| 48 | What is the vaccination status? | 1. completed 2. not completed  3. up to date 4.unknown |  |
| 49 | Is the child received vitamin A supplementation? (in the last 6 months) | 1. Yes  0. No |  |
| 50 | Had the child suffer from infections like Diarrhea, fever and Cough for the past 2weeks? | 1. Yes  0. No |  |

PART V: Food insecurity and malnutrition

| Code | Questions | Coding category | Skip to |
| --- | --- | --- | --- |
| 51 | Were you or any household member not able to eat the kinds of food you preferred because of lack of resources | 1. Yes  0. No | If no, skip  to 60 |
| 52 | If yes, how frequent? | 1. Often 2. Rarely 3. sometimes |  |
| 53 | Did you or any household member eat just a few kinds of food day after day due to lack of resources? | 1. Yes  0. No | If no skip to Q 62 |
| 54 | If yes, how frequent? | 1. Often 2. Rarely 3. sometimes |  |
| 55 | Did you or any household member eat a smaller meal than you felt you needed because there was not enough food? | 1. Yes  0. No | If no  skip  to Q.64 |
| 56 | If yes, how frequent? | 1. Often 2. Rarely 2.sometimes |  |
| 57 | Did you or any household member eat fewer meals in a day because there was not enough food? | 1. Yes  0. No | If no  skip  to Q.66 |
| 58 | If yes, how frequent? | 1. Often 2. Rarely 3. sometimes |  |
| 59 | Was there ever no food at all in your household because there were not enough resources to get more | 1. Yes  0. No |  |

PART VI: Anthropometric Measurements

| 60 | Age of the child | ___________month |  |
| --- | --- | --- | --- |
| 61 | Weight of the child | ___________kg |  |
| 62 | Height of the Child | __________cm |  |
| 63 | MUAC of the child | _  ________ mm |  |
