# Supplementary material for: Prevalence of undernutrition and its associated factors among orphans aged 6–59 months in Nekemte town, Ethiopia
Source: PLoS One. 2025 Nov 13;20(11):e0336300. doi: 10.1371/journal.pone.0336300 (PMC12614551; doi:10.1371/journal.pone.0336300)
Supplement: Attachment — Submitted filename: Responses to Reviewers first round.docx [file pone.0336300.s005.docx]

**Responses to Editor and Reviewers**

**Dear Editor,**

Thank you for your patience and for the valuable scientific contributions you made to enhance our manuscript's readability and credibility. We have addressed the comments and questions raised by the editor in detail below.

**Response to Editor,**

**Dear Editor, we appreciate your commitment and excused for the delay.**

**Comment:** Please include a rebuttal letter that responds to each point raised by the academic editor and reviewer(s). You should upload this letter as a separate file labeled 'Response to Reviewers'.

**Response:** Thank you very much. We have provided answers to each questions raised line by line which can be evidenced from the cleaned version of the manuscript. The lines and pages stated in this responses are those in the cleaned version of the manuscript.

**Comment:** A marked-up copy of your manuscript that highlights changes made to the original version. You should upload this as a separate file labeled 'Revised Manuscript with Track Changes'.

**Response:** A cleaned version of the manuscript is uploaded in the system.

Comment: An unmarked version of your revised paper without tracked changes. You should upload this as a separate file labeled 'Manuscript'.

**Response:** A tracked version of the manuscript is uploaded and found in the system.

**Comment:** If you would like to make changes to your financial disclosure, please include your updated statement in your cover letter. Guidelines for resubmitting your figure files are available below the reviewer comments at the end of this letter.

**Comments:**  When submitting your revision, address these additional requirements.

Please ensure that your manuscript meets PLOS ONE's style requirements, including those for file naming. The PLOS ONE style templates can be found at https://journals.plos.org/plosone/s/file?id=wjVg/PLOSOne_formatting_sample_main_body.pdf and

<https://journals.plos.org/plosone/s/file?id=ba62/PLOSOne_formatting_sample_title_authors_affiliations.pdf>.

**Response:** Thank you for suggesting to rearrange. The revised manuscript is rearranged according to the manuscript. This can be seen throughout the document.

**Comment**: We note that your Data Availability Statement is currently as follows: [All relevant data are within the manuscript and its Supporting Information files. Please confirm at this time whether or not your submission contains all raw data required to replicate the results of your study. Authors must share the “minimal data set” for their submission. PLOS defines the minimal data set to consist of the data required to replicate all study findings reported in the article, as well as related metadata and methods (https://journals.plos.org/plosone/s/data-availability#loc-minimal-data-set-definition). For example, authors should submit the following data:

- The values behind the means, standard deviations and other measures reported;
- The values used to build graphs;
- The points extracted from images for analysis.

Response: We apologize for any confusion. The dataset referenced in this manuscript is submitted alongside the revised manuscript. The values behind means, standard deviations and other reports, graph values and images reported in the manuscript are based on the data in the data set. It is provided in an Excel spreadsheet format, as we were informed that the minimum dataset submitted in SPSS could not be opened. This dataset has been uploaded to the system during the submission of the revised work. The age, residence and date of birth of the study participants were removed from the data set. Otherwise, other relevant data are found in the manuscript.

**Authors’ responses to journal questions**

**Comment:** Is the manuscript technically sound, and do the data support the conclusions?

**Response:** We modified the conclusions as suggested and stated it as below (page 2, line 57-60). In this section, the minor technical error was made based on the given suggestion. It is rewritten as follow:

**“Conclusions:** The study concludes that nursing students have limited knowledge and skills regarding children's environmental health. To address this gap, it is strongly recommended to integrate children's environmental health into the nursing curriculum and to encourage nursing students to enhance their understanding and improve their skills in this vital area”.

**Comment:** Has the statistical analysis been performed appropriately and rigorously?

**Response:** For this questions there was no question raised. Thus, we tried to identify mistakes in the statistical part. However, we didn’t make any changes to the statistical parts as there is noted mistake to our level of checking.

**Comment:** Have the authors made all data underlying the findings in their manuscript fully available?

**Response:** The findings tried to answer the objectives of the study and are included in the results sections, and discussed further in the discussion section.

**Comment:** Is the manuscript presented in an intelligible fashion and written in Standard English?

**Response:** We tried to improve the standard of the English language usage. This could be seen throughout the document.

**Dear Reviewers,**

Thank you for your valuable insights that have significantly improved the quality of our manuscript. We appreciate the time you dedicated to this process and your contributions to the scientific community, benefiting people around the globe. Below are our responses to the comments and questions raised.

**Response to Reviewer #1**

**Comment:** In the introduction section, line 88-91, the sentence is incomplete.

**Response:** Thank you for your great emphasis, we left to add ‘were associated with undernutrition in under five year’s old children”. Therefore, we have corrected as the following Evidence revealed that educational level, lack of employment for the head of the household, large family size, and food insecurity, insufficient food consumption, and a high rate of infectious illnesses, lack of vitamin A supplementation, poor knowledge and attitude of caregivers, and diarrhea were associated with undernutrition in under five years old orphan children (page 4, line: 101-108).

**Comment:** In the last paragraph of the introduction, the author claimed that there is no study investigating the prevalence of undernutrition in under-five orphan children in Nekemte town, but did not provide any information about the existing research in this area in other cities in Ethiopia, other countries or international data, if available. I found several similar studies conducted in Dilla (https://doi.org/10.1186/s40795-019-0295-6), Addis Ababa (https://doi.org/10.1186/s40795-021-00431-5), Gondar (10.11648/j.jfns.20140204.23), and Gambella Southwest (https://doi.org/10.1136/bmjopen-2020-045892) through a brief web search. Please review the similar studies in the introduction and provide the rationale for performing this study in Nekemte town.

**Response**: This is very important comment that enhances us to see many sources related to our article. But, as you mentioned, we only focused on the Nekemte city which is our main study area in context of the topic. We have included in the revise version of manuscript, specifically in the statement of the problem and discussion sections.

**Comment:** In the method section, the sentence in which eligibility criteria have been listed (lines 109 -110) seems incorrect. It should be revised.

**Response:** Exactly, it was a typing error. We wanted to say, orphan children 6-59 month living in Nekemte Town who are paired with caregivers or guardians were included and orphan children who were who were seriously ill and with physical disabilities as well as whose caregivers had hearing loss were excluded from the study. So, we have corrected on the manuscript with highlight text.

**Comment:** The author stated that the simple random sampling method has been applied. In this sampling method, we need a complete list of the target population members. It should be clarified where the researchers achieved such a list from. Are these data recorded in a registry system?

**Response:** The study participants were selected using simple random sampling techniques. We employed a lottery method based on the registration books to generate a sampling frame. The registry was obtained from the town youth and women' office. When two or more orphans were discovered in the same household, the younger one was chosen for the study (page 5, line: 141-144).

**Comment:** There is no information about the questionnaire used for assessing food security. Has the questionnaire been validated before in this population? Is there a scoring system for this questionnaire in order to rate the food security status of the households or identify food insecure ones? Presenting the individual questions is not that informative. And the main question is that what was the aim of assessing food security status in this study?

**Response:** Thank you very much, we assumed as if the one displayed in figure is enough to yield full information. Now we have added to the manuscript in both text and figure according to following. The number of under-five orphan children in Nekemte town is 663; out of 295 are male, and 368 are female. Sample size was calculated to get 384 as final. Data was taken from orphan children registration book in Nekemte town children, women, and youth affairs Bureau (page 5, line: 130-139).

**Comment:** In table 1, height and weight data have been presented. In the pediatric population raw anthropometric measures are not informative, please provide Z-score or percentile data

Response: Best reminder; we have not conducted food security which is to be another study. For the moment, we only focused on the locally available food and traditional eating behavior. Other questionnaires were pretested before actual data collection that kept the quality of information (Table1, page 10).

**Comment:** In tables 1-4, descriptive demographic, environmental, social, etc., variables have been presented in a descriptive manner. I suggest to add two columns to these tables comparing the variables between well-nourished and malnourished children and use appropriate tests to explore the statistical significance of differences.

**Response:** Thank you for relevant information. We did not use in the table because of our dependent variables are categorized into four: normal, underweight, wasting and stunting. **Comment:** In table 1, there are some repetitive rows, like “Occupation of the caretakers” or “Whom did the child lose”. Please revise them.

**Response:** We used the two phrases for different information. For example,” Occupation of the caretakers” speaks about what do the care takers do as regular activity to survive. The second “Whom did the child lose” wants to say from the parent, did a father or a mother die exposing them be orphan? So, let it be understood in such manner.

**Comment:** The second table in the text has been labeled as table 1 again.

Response: Exactly, thank you really. That was a typing error again, we have corrected it now in revised manuscript

**Comment:** The results of table 5 show that, not taking vitamin A supplements decrease the probability of being stunted. This is an unexpected result, and no explanation about that has been provided in the discussion section.

**Response:** Thank you very much. It was rewritten as “The study found that orphans who did not receive vitamin A supplements had a 41% higher chance of developing stunting than those who did (AOR: 0.59, 95% CI: 0.371-0.94)” (PAHE 15, LINE: 281 & 282). Absence of the vitamin A exposes the child for stunting.

**Comment:** The whole manuscript text needs extensive English language editing

**Response:** We tried to reach for the English language accuracy.

**Responses to Reviewer #2**

**Comment:** Clarify the punctuation and eligibility criteria, with a focus on the specific target groups.

**Response:** Thank you a lot. We have revised the punctuation and grammar error in manuscript that will see it now. On eligibility criteria we have corrected as “orphan children 6-59 month living in Nekemte Town who are paired with caregivers or guardians were included and orphan children who were who were seriously ill and with physical disabilities as well as whose caregivers had hearing loss were excluded from the study”.

**Comment:** Provide more details on sample size determination, including specifications for the non-response rate.

**Response:** You reminded as important point. The sample size was calculated for both first and second objectives based on available references. Accordingly, we have calculated for the first objective using a study done in Addis Ababa City (Teferi H, 2021) on prevalence of stunting, wasting and underweight that yield 349 as maximum from stunting prevalence compared to others. For the second objective, we used same article showing different significant variables that yield 334 as a maximum sample size based on Vitamin A supplementation. Therefore, from both sample size, 349 taken as the maximum and 10% non-response rate was added to be the final **384** as described in figure in revised form of manuscript.

**Comment:** Ensure appropriate referencing for the operational definitions of stunting, wasting, and other terms.

**Response:** Okay, thank you! Now we have corrected in revised manuscript

**Comment:** The report lacks proper definitions or explanations of the abbreviations used, such as FGFS.

**Response:** Nice reminder, we crossed over it while searching abbreviations in the body texts. We mean to say “Fibroblast Growth Factors” for FGFs.

**Response:** Thank you very much! But, it is good if you as any specific areas we poorly interpreted to reach at it as easy as possible. Anyhow, we will try to cross check all parts.

**Comment:** The interpretation of the analysis needs to be addressed more thoroughly. In some cases, interpretations are provided without corresponding information in any table. Recheck the analysis to identify whether additional information should be included or if irrelevant statistics need to be removed

**Response:** Yes, we mistakenly made some error on typing; now we have corrected in revised manuscript.

**Comment:** Tables in the manuscript need to be properly formatted with accurate headers and estimates.

**Response:** Thank you. We modified in the revised manuscript.

**Comment:** In the results section, there are many variables presented in the tables that are not interpreted or discussed. This should be reviewed for completeness

**Response:** Essential. now we have corrected in the revised manuscript that you will come across.

**Comment:** Revise manuscript according the journal style, English language usage and discussion reconstruction

**Response:** We tried to improve our drawbacks which could be seen from the revised manuscript.. We tried to submit the manuscript in texts, table and figure separately. The remained will be corrected again. Thank you again we tried to revise based on the given comments and our reviews. If we miss any part, we are ready to accept your feedback more.
