# Supplementary material for: Prevalence of undernutrition and its associated factors among orphans aged 6–59 months in Nekemte town, Ethiopia
Source: PLoS One. 2025 Nov 13;20(11):e0336300. doi: 10.1371/journal.pone.0336300 (PMC12614551; doi:10.1371/journal.pone.0336300)
Supplement: Attachment — Submitted filename: Response to Reviewers.docx [file pone.0336300.s006.docx]

**Responses to Editors and Reviewers**

Dear Editor and reviewers,

Thank you very much for sharing your invaluable resources with us: your time, deep knowledge, and expertise, which have greatly enhanced our understanding of undernutrition in orphans aged 6 to 59 months. We sincerely appreciate the significant effort you invested in shaping our manuscript to improve its quality. We have learned a great deal from your comments and have made corrections in the areas identified for improvement. We are grateful for your insights and have accepted the suggestions provided.

**Editors’ comment**

**Dear Authors, Please kindly address all the comments and submit the revised version. Specifically, you have to address the sample size determination issues clearly considering the three primary outcomes. In addition. I am not clear with 348 and 384?**

Response: Thank you very much for great concern. It is a clear mistake. We corrected it as follow: We estimated the sample size using the single population proportion based on a 5% margin of error, a 95% confidence interval and the prevalence of stunting from the study conducted in Addis Ababa (34.8%) among orphans aged 6-59-months(9). Based on these assumptions, we obtained 349 samples. By adding a 10% for the nonresponse rates, the final sample size was 384 (page 9, line 130-133).

Editorial comments

**Comment 1: Abstract line 54 U5C, is not standard form of writing, write fully and even in the main document.**

Response: Thank you. We removed from this section also suggested by reviewer #3.

**Comment 2: Operationalized orphan in your study context**

Response: Thank you. We provided the definition of an orphan in the revised manuscript under subheading of operational definitions as “Orphan: is a child who has lost one or both parents through death (Page 6, line 144).

**Comment 3. Editorial issues from line 240 and 252, 254, 2654. In the factor table-5 sub-section you mentioned wasting two times and you missed underweight part5.**

Response: Thank you very much. These parts are corrected throughout the document.

**Comment 4: How do you approach the data collection process, is that school, community house to house or other settings clearly state in the method part**

Response: The study was community based. We introduced in the methods section under study setting, population and period. We apologize for not mentioning it in the methods section.

**Responses to Reviewer #2:**

**Comment 1: Sample size inconsistency: The manuscript states that the sample size was determined to be 384, accounting for a non-response rate, yet the final number of participants used in the analysis was 373. Please provide a clear explanation of the sample size calculation and the reason for this discrepancy. This is a critical methodological component of the study and must be transparent.**

Response: Thank you very much for great concern. It is a clear mistake. We corrected it as follow:

We estimated the sample size using the single population proportion based on a 5% margin of error, a 95% confidence interval and the prevalence of stunting from the study conducted in Addis Ababa (34.8%) among orphans aged 6-59-months(9). Based on these assumptions, we obtained 349 samples. By adding a 10% for the nonresponse rates, the final sample size was 384 (page 9, line 130-133).

**Comment 2: Statistical notation consistency: Please maintain consistency in reporting descriptive statistics. Use the format ‘Mean ± SD’ instead of ‘Mean and SD’. Also, when reporting frequencies, please use the format ‘Frequency (n)’ to improve clarity and consistency.**

Response: We reorganized it as follow: The mean and standard deviation (Mean ±SD) of age, weight and height of the orphans was 33.78±17.8 SD months, 12.99±3.25 kg, and 88.22 ± 14.83cm, respectively. Response: We modified the points in the revised manuscript (page 9, line232-240).

**Comment 3: Unclear software-based reference before table: There is a reference like “Error! Reference source not found.” before Table 1. This appears to be a software-generated location or remove it if not relevant.**

Response: Great. It is modified using the system (Page 9, line 240-242).

**Comment 4: Table 2 format issues (WFA, HFA, WFH): The structure of Table 2 requires correction, particularly in the presentation of WFA, HFA, and WFH. Please ensure the variables are clearly defined, appropriately labeled, and aligned with standard anthropometric reporting formats.**

Response: Thank you. The dependent variables (underweight, height and weight) are redefined in the methods section under the subheading operational definitions. The abbreviated variables WFA, HFA, and WFH in the table one are removed as it is similar to the result findings in the figure 1(page 9, table1 and page 14, 275-278).

**Comment 5: Table mislabeling: Table 2 is mistakenly labeled as Table 1. Please correct the numbering to maintain logical sequence throughout the manuscript.**

Response: We modified this table using the system generated and correctly ordered in the revised version of the manuscript (page 11).

**6. Percentage total inconsistencies (Table 4): Percentages must always sum to 100% within their respective categories. For follow-up questions that apply only to a subset of respondents (i.e., those who answered ‘Yes’), ensure that the “n” reflects only that subgroup rather than the total sample.**

Response: Thank you very much for your suggestion. We made several corrections regarding the frequencies and percentage for table 4 as suggested (Table 4, page 12 and 13).

**7. Missing frequency data for stunting and wasting in Table 1: Frequencies for stunting and wasting are not included in Table 1. As these are key outcome variables, please ensure that their frequencies are reported clearly alongside their corresponding percentages.**

Response: Thank you very much. It was wrongly stated. However, since the correct one is mentioned under the subtopic prevalence in figure one, we prefer to use it (Page 14, line 275-278).

**Responses to Reviewer#3**

**Comment 1: Socio-Demographic Characteristics of Orphans and Their Caregivers in Nekemte Town, Ethiopia, 2023--- provide the upper age limit in months for the second child age category (≥24 months).**

Response: Thank you for your informative comment. We takes a look at and edited as you suggested throughout the tables and documents.

**Comment 2: Health status, environmental, behavioral, social and legal services, knowledge and attitude related characteristics of orphan children in Nekemte town, Ethiopia, 2023--- This table should be labelled at Table #2. In addition, the title refers to “attitude-related” characteristics but I did not see any data in this table related to attitudes. Please explain, revise as appropriate.**

Response: Thank you. We revised using the table labels using the insert caption. However, the attitude of the caregivers typed error. In the revised version of our manuscript, we have removed it (Table 2, Page 11& 12).

**Comment 3: Table 3—explain what is meant by “flat foods”**

Response. Thank you very much. It is typing error and corrected to ‘fat’ (Table3, page 12 &13).

**Comment 4: Please review and edit for clarity the track changes for the summary of results in the discussion section in lines 354 to 360 as Addis Ababa is mentioned multiple times.**

Response: Alright. There is a repetition. It modified in its revised version of the manuscript (page, line).

**Comment 5: Some suggestions in the conclusion section go beyond the data collected in this study. Lines 463-464 in the Conclusion section should be edited for clarity and grammar (e.g., change “frequency to prevalence” and revise “marked public health significance”: Conclusion: Overall, the frequency of stunting, wasting, and underweight among orphaned children was marked public health significance**

Response: It is revised as follow “It is crucial to enhance the knowledge and communication skills of healthcare workers and caregivers regarding infant and young child feeding practices and vitamin A supplementation for children under five years of age. Additionally, improving supervision of orphans by legal bodies is essential for better health outcomes.” (page 2, line 52-56).

**Comment 6: The article is presented in an intelligible fashion and is written in Standard English. The article is written in Standard English that is acceptable. There are some sentences and definitions that lack clarity. For example:• The introductions says: “According to Ethiopia's malnutrition trend, the prevalence of stunting has reduced from 58% to 38% and that of underweight has fallen from 41% to 24% over the last fifteen 15 years from 2000. However, the prevalence of wasting has marginally declined during the previous 15 years, from 12% to 10% (4,75 5).” When referring to the previous 15 years, the reader would anticipate this time period would be between 2010 and 2025. However, these two sentences appear to refer to 2000-2015.**

Response: Thank you very much. We corrected 15 years to 2000-2015 years (page 3, line 70-72).

**Comment 7: The definitions of food security and insecurity in the Methods section do not appear to conform to global standardized definitions. For example, they are not aligned with the FAO or FANTA definitions.**

Response: Thank you very much. We revised the definition of food security and insecurity by replacing the operational definition suggested for this study and cited (page 6, line 157-162).
