# Supplementary material for: Prevalence of undernutrition and its associated factors among orphans aged 6–59 months in Nekemte town, Ethiopia
Source: PLoS One. 2025 Nov 13;20(11):e0336300. doi: 10.1371/journal.pone.0336300 (PMC12614551; doi:10.1371/journal.pone.0336300)
Supplement: Attachment — Submitted filename: Response_to_Reviewers_auresp_4.docx [file pone.0336300.s007.docx]

**Response to Editor and Reviewers**

Dear Editor and Reviewers,

Thank you for your time and the valuable scientific contributions you made to improve the quality of our manuscript. We appreciate your insights and suggestions, which have significantly enhanced our work.

**Please include the following items when submitting your revised manuscript:**

**Comment:** A rebuttal letter that responds to each point raised by the academic editor and reviewer(s). You should upload this letter as a separate file labeled 'Response to Reviewers'.

**Response:** A letter responds to each point raised raised by the academic editor and reviewers is provided and attached in the online submission with the file name labelled as ’Response to Reviewers.

**Comment:** A marked-up copy of your manuscript that highlights changes made to the original version. You should upload this as a separate file labeled 'Revised Manuscript with Track Changes'.

**Response:** A manuscript with track version is submitted with the file name labelled as 'Revised Manuscript with Track Changes'.

**Comment:** An unmarked version of your revised paper without tracked changes. You should upload this as a separate file labeled 'Manuscript'.

**Response:** Cleaned version of the manuscript is submitted with the file name labelled as ‘Manuscript’.

**Journal Requirements:**

**Comment:** If the reviewer comments include a recommendation to cite specific previously published works, please review and evaluate these publications to determine whether they are relevant and should be cited. There is no requirement to cite these works unless the editor has indicated otherwise.

**Response:** We did not encounter this kind of recommendation.

**Comment:** Please review your reference list to ensure that it is complete and correct. If you have cited papers that have been retracted, please include the rationale for doing so in the manuscript text, or remove these references and replace them with relevant current references. Any changes to the reference list should be mentioned in the rebuttal letter that accompanies your revised manuscript. If you need to cite a retracted article, indicate the article’s retracted status in the References list and also include a citation and full reference for the retraction notice.

**Response:** "We did not include any retracted papers in this manuscript."

**Reviewers Comments**

**Comment:**  Great correction, I appreciate it. However1. In Table 4, for the question 'Did you worry about your family not having enough food?', there is a skip pattern issue that has not been corrected accordingly.

**Response**: Thank you very much for your attention, as it is greatly appreciated. We identified an error, which we corrected by calculating the percentage of the 190 respondents who answered 'yes' to the question, 'Did you worry about your family not having enough food?' (see Table 4).
**Comment:** The other corrections seem fine, but there are still some spacing inconsistencies throughout the manuscript. Please review and correct these carefully.

**Response:** We have identified several inconsistencies in our manuscript and have made the necessary corrections. This is found throughout the manuscript.
